# Typhoid Fever

**Published:** 1908-01

**Authors:** W. A. Mathews

**Affiliations:** Hawkinsville, Georgia


### *TYPHOID FEVER.

BY W. A. MATHEWS, M. D., HAWKINSVILLE, GA.

Typhoid fever is an acute, infectious febrile disease, characterized by inflammation and ulceration of pyers patches, the solitary glands of the small and large intestines and the mesenteric glands, while often the spleen and other organs of the body become infected.

Most writers mention several varieties of the disease; the abortive, mild, severe, hemorrhagic, ambulatory, renal, pneumonic and that of children and old age, etc. It will serve our purpose, however, to class the disease in two types, viz: the mild or non-malignant, and the severe or malignant. The symptoms of typical cases of typhoid fever are so striking that a layman can make a diagnosis, yet we have atypical cases in which the best diagnosticians can not arrive at a definite conclusion.

The predisposing causes are autumn season, adult life and personal susceptibility, while the exciting cause is the introduction into the system of the bacillus typhosus, or bacillus of Eberth, (discovered by Eberth in 1880).

From the intestinal discharge of person suffering with the disease is the principal source of contagion, and through the drinking water is the chief mode of transmission, although it may be contracted through the milk and other articles of raw diet.

The course of infection is probably as follows: When contaminated water, milk or food enters the stomach, if there is a normal amount of hydrochloric acid present, they are at once destroyed, but if there is a deficiency of this acid, they pass out of the stomach unharmed into the intestines, where conditions are favorable for their development, they find their way into the lymph glands and from there, they, and the poison they produce, enter the blood and are carried to the different organs and tissues of the body.

The stage of infection varies in its duration, but is generally supposed to be from fifteen to twenty days. The onset is so gradual that often a physician is not consulted until the disease

*Read before the Ocmulgee Medical Association, Eastman, Ga., July 16th, 1907.

is well established, and the fever is found to be as high as 102 or 103, so it is difficult to definitely fix the time when the disease actually begins. Flint reckons from the time when the patient takes his bed, but this is unsatisfactory for the reason that, some people have a horror of going to bed when sick and presistently stay up, while others take their bed for the most trivial causes, besides, we have the mild and ambulatory types, in which the patients do not remain in bed during the entire course of the disease. My custom is to obtain from the patient, or some member of the family, as near as possible the date when fever first began and reckon from that time.

As I have said, in typical cases of typhoid fever, the symptoms are so suggestive that it is not difficult to make a diagnosis. We have the typical prodromal stage, expressionless countenance, frontal headache, epistaxis, heavily coated tongue in the center with clean, red edges, the rose spots, the peculiar odor of the patients clothes and bed linen, the step like elevation of the temperature, the dicrotic condition with relative slowing of the pulse, often with temperature 10 3or 104 the pulse remains constantly between 90 and 110, we have the tympanities with tenderness and gurgling in the right illiac fossa, diarrhœa with its typical odor and pea soup appearance, separating in two layers, an upper thin fluid with lower more consistent mass.

All of the above symptoms go to make a typical case of typhoid fever, yet we have cases of fever continuing from day to day, and at times for weeks, with none of the above symptoms pronounced and apparently without any definite cause.

In such cases, we should, by careful examination and with the aid of the family history, eliminate the probable existence of other diseases simulating typhoid, and apply the widal agglutination test.

It has been said by a clever Diagnostician, (Herrick) that "typhoid fever is not only an imitator of diseases, but many diseases imitate typhoid." In pyemic conditions, especially the cryptogenic variety, where the source of infection is deeply seated, we have many positive typhoid symptoms. We have the malaise, headache, furred tongue, the nervous phenomina and often the rash; to differentiate the disease, we have chilly periods during the entire time of illness with quick elevations of temperature, the absence of abdominal and especially the illiac symptoms. We often have pains at the points of infection, with absence of the relative slowness of the pulse, but on the contrary, they are quick and irregular. These points of differences are well to remember, as it is humiliating to pronounce a deep seated abscess, typhoid, and treat it as such for weeks, when finally the true condition is revealed to the family, friends and neighbors. In miliary tuberculosis we have often pronounced typhoid symptoms with a striking absence of tubercular symptoms, which renders a differential diagnosis difficult. In such cases, the widal test should at once be resorted to, and the sputum and urine examined for tubercle bacilli. The slightest deviation of the sounds at the apex of the lungs attended by cyanosis, with pulse ranging from 120 to 130 is in favor of tuberculosis and should be well considered in arriving at a diagnosis. Since the two diseases have been so often taken one for the other, and so much depends upon the proper treatment of each in its early stage, and in view of the fact that, it is not always convenient to have the widal test made, or the sputum examined, I mention a simple test that any physician can make, which will aid materially in differentiating the two diseases. Draw about half drachm blood from the patient into a small test tube and set it aside for twenty-four hours; if the patient is suffering from typhoid fever, there will be scarcely any serum formed and the clot of coagulated blood will be found closely adherent to the sides of the tube; while if it is tuberculosis, the clot will be retracted and there will be an abundant formation of serum.

In this section of the country, the remitent type of malarial fever is more often taken for typhoid than any other disease and vice versa.. There are a few marks of distinctions, however, which are well to note; in malaria, the remitent character of the fever is noticable from the onset; ther is a pale, flabby tongue, not trimulous, and the patients become anaemic early in the disease. The pulse is not dicrotic, but is relatively faster than in typhoid, the abdominal symptoms are not so marked, while the illiac symptoms and rose spots are absent.

There are two other, more definite diagnostic points which in obscure cases should be made use of. The failure to obtain the widal reaction, which, however, is not always obtained in typhoid, especially in the early part of the disease, but if you do get it, the diagnosis is definitely settled. Next is the administration of quinine in large doses for from four to six days, which is my routine practice when called to any case of fever of a doubtful or obscure origin. If, after giving quinine in large doses, twenty to thirty grains a day for five consecutive days, in a case of fever where other causes have been eliminated and the fever continues, I think we are justified in making diagnosis of typhoid or para typhoid and treat the case accordingly. There are other diseases simulating typhoid, such as tubercular peritonitis, ulcerative endocarditis, salpingitis on the right side, catarrhal enteritis in children, influenza, uremia, typhus and relapsing fevers, but the time allotted me for this paper will not permit me to go into the differential diagnosis of them all.

I shall give briefly the pathology of the disease, that I may be justified in the line of treatment I shall advocate.

The lesions of typhoid fever are found principally in Peyer's patches, the solitary glands of the small and large intestines and the messenteric glands, while the spleen is usually somewhat enlarged and softened.

The process may be divided into four well defined stages, viz:

1st., Infiltration or swelling. 2nd., Necrosis or softening and sloughing. 3rd., Ulceration. 4th., Cicatrization.

During the first stage, the glands are elevated above the surrounding mucous membrane, there is a hard infiltration and proliferation of cellular elements. On account of this hardening of the glandular tissue, circulation is inhibited and produces a softening of the glands, followed by sloughing. This occurs about the end of the first week, and is the begining of the second stage, which continues through the second and third weeks.

At the beginning of the disease, there may be only a few glands infected, but as they begin to break down and slough, the bacilli, which have been rapidly multiplying, are liberated, seek and infect other glands. During this stage, the symptoms usually become more pronounced and the condition more grave. During the third stage, we have the period of ulcers which are formed by the necrosed tissue, beginning at the edges and gradually separating from the healthy tissue, leaving ulcers of various sizes with a smooth base of submucous, muscular or peritoneal tissue, dependent upon the depth to which the neurosis had gone.

The healing of these ulcers leaves slightly depressed scars of cicatricial tissue. While the four processes are divided into periods, it is not to be understood that they are separate and distinct as to the time in which they exist, but after the first week we probably have the four conditions in force at the same time, until the disease is checked.

The duration and severity of the disease depends probably, to some extent, upon the malignancy of the infection in some cases and in certain epidemics, also the idiosyncracy of the patient, but in my opinion, it depends more than all upon the treatment.

As soon as a physician makes a diagnosis of typhoid fever, he should carefully inspect the premises for the source of infection, for the protection of the other members of the household. The patient should be isolated to as great as possible, and the nurse and assistants should be given specific instructions as to the handling of the linen, disinfecting the excreta, room, stools, closet, etc. A light, but nourishing diet should be prescribed, for which I have no special list. In severe cases, I restrict the diet to liquids only, such as milk, peptonoids, barley, or rice water, beef juice, broth, etc., while in mild cases, I allow them to take thin suops, raw eggs baked apples, etc., in addition to the liquids. It is of very great importance to see that the foods taken are digested and assimulated, as the digestive functions are impaired during fever. Patients should be encouraged to take plenty of drinking water, as free diuresis aids in eliminating poison from the system. After the first week, except in mild cases, patients should not be allowed to assume the sitting posture.

The temperature should be taken about the same time morning and evening. A sponge bath should be given each day, using massage to stimulate the peripheral circulation, the integument and superficial muscles. There should be a daily change of linen of both bed and patient.

Upon the details of the general management of a case of typhoid fever, I believe all physicians practically agree, but when it comes to the medicinal treatment, harmony takes its flight and the profession stands divided, advocating two extremes; some contending for the expectant method of treating the symptoms as they arise, letting the disease or the underlying cause alone, except if possible, guiding it safely through its inevitable course; others advocate the Brand or cold water treatment, depending almost entirely upon the baths to control the symptoms and cure the disease,; while there is still another class who use antiseptics and eliminants, with the conviction that by this method, the disease is either shortened or aborted.

I consider it entirely impractical to carry out the Brand method in general practice, even if there were sufficient virtue in it to justify the claims made by its advocates. The expectant plan may be adopted in mild cases, but in severe or malignant types of the disease, statistics abundantly prove this method to be inadequate. I am a firm believer in the antiseptic and eliminative plan upon general principles, as well as to take care of the symptoms and complications as they arise. During the first week, or during the stage of induration, I give at least two good courses of calomel, such as I would in billious or malarial conditions. This is to thoroughly cleanse the system and fortify nature against the effects of the disease. I also give during this period, large doses of quinine to make sure of my diagnosis, and as a specific to remove malarial complications should any exist. I also give trional and caffeine citrate when necessary to control painful or nervous conditions. At the beginning of the second week, I put my patient on a capsule every three hours, containing guaiacol carbonate 3 gr. menthol ½ gr. and podophilin one twelfth gr., also a teaspoonful of iodinized emulsion, which contains to each drachm, spiritis turpentine 3½ min., tr. iodine 1¾ min., oil peppermint one-eighth min. and oil gaultheria ¼ min., prepared in an emulsion with elixir pepsin comp., by the Dawson Pharmacal Co., of Dawson Springs, Ky. I consider this the most e-f fective, as well as palatible preparation of its kind on the market. Every four or five days, I substitute ½ gr. calomel for the menthol in the capsule and continue it for six or eight doses, or until the glandular system is thoroughly aroused. I also give occasionally a dose of castor oil, or an enema of tepid water with a long, soft tube, washing out the entire colon. I continue this line of treatment through the entire course of the disease with slight modifications to meet the symptoms or complications as they arise.

The treatment is based upon the idea that as soon as the first infected glands begin to slough there is turned loose in the intestines increased numbers of typhoid baccilli to attract unaffected glands, thereby continuing the disease. The action of these germs produces a violent poison that is being constantly taken up by the blood and carried to every part of the system, causing elevation of the temperature, with all the attendant symptoms. The sloughing of the glands sets up a putrefactive process, which within itself, is an irritant and should be removed.

The purpose of the antiseptic and eliminative treatment is, to inhibit the activity of the bacilli, to neutralize the toxins and to remove these with the putrefactive elements, from the intestinal canal as rapidly as possible. As evidence that this can at least be partially accomplished when we administer the remedies, the character of the stools change from an ill-smelling liquid, to one with scarcely any odor at all except that of the drug, we have a lower temperature with a decided amelioration of the other symptoms, with fewer complications and less tendency to relapse. The high fever is not the predominating, nor consitive factor in typhoid conditions, but is a result of the morbid condition, and is primarily due to the influence of the infected blood upon the nervous system, hence under this treatment we do not have to give antipyretics, nor use cold baths for the reduction of the temperature.

The low mortality under the eliminative treatment, as compared to that of other methods, is one of its strongest points of recommendation.

A canvass a few years ago by Dr. H. G. McCormack, of the Williamsport Hospital, Williamsport, Pa., of sixteen of the principal hospitals of the country, shows that out of 1998 cases treated by the expectant and Brand methods, there were 265 deaths, about 13 per cent.

At the same time there were treated at the Williamsport Hospital 408 cases by the antiseptic and eliminative plan, with only 22 deaths, about 5.4 per cent, being a reduction in the mortality of over fifty per cent.

Dr. Virgil T. Hubbard, of Atlanta, who is considered among the best authorities on typhoid fever conditions, reports over one hundred cases treated with only one death, and that the patient had chronic Bright's disease in addition to the typhoid.

My results obtained from the treatment, for the past seven years have been exceedingly satisfactory, having lost only two cases and with them the nursing was extremely poor, so much so that I could not have the treatment administered or directions followed to any degree of satisfaction.

The above observation and experience of those who use the treatment, would indicate that it is capable of the following:

1st: It inhibits the activity of the typhoid bacilli.

2nd: That it prevents to a degree the congestion of the glands of the intestines.

3rd: That it stimulates the glandular system and helps nature to combat the disease.

4th: That it removes from the intestinal tract the loose bacilli, gasses and other products of putrefaction, thereby lessening the danger of hemorrhage and perforation.

5th: That it neutralizes typhotoxin, causing a reduction of fever, lessening delirium, stupor, and modifies various other symptoms.

6th: That since it lessens the intestinal involvement, it furnishes an abortive treatment and reduces to a minimum the dangers of complications and relapses.

